# Therapeutic Potential of Novel Twin Compounds Containing Tetramethylpyrazine and Carnitine Substructures in Experimental Ischemic Stroke

**DOI:** 10.1155/2017/7191856

**Published:** 2017-12-13

**Authors:** Ziying Wang, Zhuanli Zhou, Xinbing Wei, Mingwei Wang, Bi-Ou Wang, Yanan Zhang, Xiaoting He, Yu Sun, Xiaojie Wang, Mingcheng Sun, Yan Zhang, Xiaowei Gong, Fan Yi

**Affiliations:** ^1^Department of Pharmacology, School of Basic Medical Science, Shandong University, Jinan 250012, China; ^2^Key Laboratory of Neuroprotective Drug Discovery of Shandong Province, Jinan 250012, China

## Abstract

Although studies have seen dramatic advances in the understanding of the pathogenesis of stroke such as oxidative stress, inflammation, excitotoxicity, calcium overload and apoptosis, the delivery of stroke therapies is still a great challenge. In this study, we designed and synthesized a series of novel twin compounds containing tetramethylpyrazine and carnitine substructures and explored their therapeutic potential and mechanism in stroke-related neuronal injury. We first screened the neuroprotective effects of candidate compounds and found that among the tested compounds, LR134 and LR143 exhibited significant neuroprotection as evidenced by reducing cerebral infarct and edema, improving neurological function as well as blood-brain barrier integrity in rats after cerebral ischemia/reperfusion injury. We further demonstrated that the neuroprotective effects of compounds LR134 and LR143 were associated with the reduced inflammatory responses and NADPH oxidase- (NOX2-) mediated oxidative stress and the protection of mitochondria accompanied by the improvement of energy supply. In summary, this study provides direct evidence showing that the novel twin compounds containing tetramethylpyrazine and carnitine substructures have neuroprotective effects with multiple therapeutic targets, suggesting that modulation of these chemical structures may be an innovative therapeutic strategy for treating patients with stroke.

## 1. Introduction

Stroke is one of the leading causes of death and acquired disability in the world. Although studies have seen dramatic advances in the understanding of the pathogenesis of stroke such as oxidative stress, inflammation, excitotoxicity, calcium overload, and apoptosis, the delivery of stroke therapies is still a great challenge. One of the major reasons is that many current therapies specifically focus on a single pathogenic factor which are not efficacious clinically. Therefore, it is essential to expand the narrow repertoire of therapeutic applications and develop new therapeutic agent candidates with multiple therapeutic targets in the vicious cascade of stroke [1].

Blood-brain barrier (BBB) is considered the main obstacle that prevents most systematically administrated drugs from entering the central nervous system (CNS), which, to some extent, accounts for the great challenge in stroke therapy [2]. L-carnitine, an endogenous compound, is responsible for energy production via transporting long-chain free fatty acids from the cytosol to mitochondria for *β*-oxidation. In addition, L-carnitine and its derivatives have high efficiency of transmembrane transport properties via certain transporters, such as organic cation/carnitine transporter (OCTN) 2, OCTN3, and amino acid transporter B (ATB), which are particularly helpful for the transportation of drugs and their metabolites directly to the target sites [3]. Studies have demonstrated that in the central nervous system, L-carnitine can be accumulated in neuronal cells. Compared with hydrophilic L-carnitine, acetyl-L-carnitine is much easier to go through the BBB [4]. Therefore, we prepared a series of novel carnitine esters by the introduction of a variety of hydrophobic groups into the primary structure of L-carnitine to improve the liposolubility, which is propitious to the drug delivery function.

Tetramethylpyrazine (TMP), a biologically active ingredient derived from the traditional Chinese herbal medicine *Ligusticum chuanxiong* Hort, was demonstrated to possess potent effects of thrombolysis, neuronal, and vascular protection [5, 6]. However, the characteristics of weak organic base and short half-life of TMP limit its clinical application. Thus, 2-hydroxymethyl-3,5,6-trimethylpyrazine (HTMP), one of the most active metabolites of TMP which processes longer half-life and has similar beneficial effects as TMP, becomes a promising candidate instead of TMP. The lipophilic prodrug of HTMP, 2-hydroxymethyl-3,5,6-trimethylpyrazine palmitate, was further demonstrated to improve the systemic availability and efficacy of HTMP [7].

In this study, on the basis of the potential neuroprotective effects of HTMP and the drug delivery property of carnitine esters, we designed and synthesized a series of novel twin compounds through integrating HTMP and different carnitine esters into one molecule based on structural combination principles [8]. Our studies showed that these new compounds exhibited great neuroprotective effects on experimental ischemic stroke, which were associated with the reduced inflammatory responses and NADPH oxidase- (NOX2-) derived oxidative stress and the protection of mitochondrial structure and function accompanied by the improvement of energy supply.

## 2. Materials and Methods

### 2.1. Chemicals

**Figure 1:**
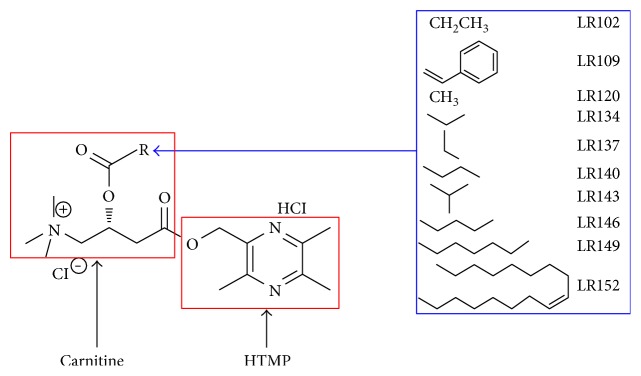
The chemical structures of 2-hydroxymethyl-3,5,6-trimethylpyrazine-carnitine ester twin compounds.

2-Hydroxymethyl-3,5,6-trimethylpyrazine-carnitine ester twin compounds and their precursors (Figure 1) used in this study were synthesized, purified, and characterized by the Key Laboratory of Neuroprotective Drug Discovery of Shandong Province (Patent number ZL 200810014211.5). The purity of the tested candidate compounds is more than 99%, analyzed by high-performance liquid chromatography (HPLC). Candidate compounds were dissolved in sterile PBS, and an equivalent amount of PBS was added to a sham-operated or model control group as a vehicle.

### 2.2. Animals

Male Sprague-Dawley rats (10 to 12 weeks old; weighing 300 ± 20 g) were obtained from Vital River Inc. (Beijing, China). All animals had unrestricted access to food/water, and all animal protocols were approved by the Institutional Animal Care and Use Committee of Shandong University and conducted in accordance with the National Institutes of Health (NIH) Guide for the Care and Use of Laboratory Animals.

### 2.3. Model for Transient Focal Cerebral Ischemia and Treatment

Transient middle cerebral artery occlusion (MCAO) was induced in male Sprague-Dawley rats as described [9]. In brief, animals were fasted overnight but were allowed free access to water. After anaesthesia with intraperitoneal injection of pentobarbital sodium (30 mg/kg), unilateral MCAO was performed by inducing a suture with 0.26 mm diameter and 0.36 mm round tip (Sunbio Biological Technology Co., Ltd., Beijing, China) into the internal carotid artery. A successful occlusion was indicated by a decrease in the regional cerebral blood flow (rCBF) to <20% of the baseline by transcranial laser Doppler (Perimed, Jarfalla, Sweden) measurement in the area of the cerebral cortex supplied by the MCA. After 2 hours of MCAO, the suture was carefully removed to restore blood flow under anaesthesia and reperfusion was confirmed by an immediate increase in rCBF. The representative results for rCBF detection were shown in Figure S1 in Supplementary Material. During and after surgery, rectal temperature was controlled with a homeothermic blanket and kept at 37°C until the complete recovery of the animal from anaesthesia. Twenty-four hours after reperfusion, the rats were deeply anaesthetized and then decapitated. The sham-operated groups were subjected to the same procedure except for occlusion of the MCA. Animals received a single intravenous injection via tail vein of the tested compounds, vehicle, or positive control drugs at different time points before or after MCAO.

### 2.4. Neurological Function and Infarct Volume Assessment

Twenty-four hours after reperfusion, the rats were scored for neurological deficits according to the 5-tiered neurological scoring system. A score of 0 reflects that the rat had no observable neurological deficit, 1 indicates that the rat failed to extend contralateral forepaw fully, 2 indicates that the rat was circling to a direction contralateral to infarct when walking, 3 indicates that the rat was falling in a direction contralateral to infarct when walking, and 4 reflects that the rat could not walk spontaneously and had a depressed level of consciousness [10]. Infarct volume was determined by 2,3,5-triphenyltetrazolium chloride (TTC; Sigma-Aldrich, St. Louis, MO, USA) staining [11]. Six coronal sections were stained with 2% TTC for 30 minutes at 37°C in the dark. Infarct volume was calculated as the thickness of the section multiplied by the total infarction areas (white part) in all brain sections. In order to correct the deviation from postischemic edema in the infarct area, an edema index was calculated by dividing the total volume of the hemisphere ipsilateral to MCAO by that of the contralateral hemisphere. The accurate infarct volume adjusted for edema was calculated by dividing the infarct volume by the edema index. The result of the ischemic volume was expressed as a percentage of the contralateral side [12].

### 2.5. Blood-Brain Barrier (BBB) Permeability

BBB permeability was evaluated by the Evans blue extravasation method. Evans blue (2% in saline, 4 mL/kg; Sigma-Aldrich) was given intravenously at the onset of reperfusion. Twenty-four hours after reperfusion, rats were transcardially perfused with saline to remove the intravascular dye. Each hemisphere was sectioned and photographed, then homogenized in 50% trichloroacetic acid and centrifuged. The supernatant was measured for absorbance at 620 nm by a full-wavelength Varioskan Flash (Thermo Electron Corporation, USA). The results were expressed as *μ*g/g tissue calculated against a standard curve [13].

### 2.6. Morphological Examinations

Hematoxylin and eosin (H&E) and 0.1% cresyl violet staining (Nissl staining) methods were used to determine the neuronal morphology in the cerebral cortex as described previously [14, 15]. To examine the ultrastructural changes in neurons and mitochondria, ultrathin sections were obtained with an ultramicrotome (UltraCut-UCT, Leica, Austria); after being stained with 2% uranyl acetate and lead citrate, samples were observed under a Zeiss EM109 (Carl Zeiss Inc., Thornwood, NY, USA) transmission electron microscopy as previously described [16].

### 2.7. Terminal Deoxynucleotidyltransferase- (TdT-) Mediated Deoxyuridine Triphosphate (dUTP) Nick End Labeling (TUNEL) Assay

Cells in the paraffin-embedded brain sections were detected by an in situ cell death detection kit, TMR red staining, according to the manufacturer's instructions (Roche Diagnostics GmbH, Germany) as described [17].

### 2.8. RNA Extraction and RT-PCR

Total RNA was isolated from brain tissue and cells by using the TRIzol reagent (Invitrogen) as described previously [9]. The mRNA levels for target genes were analyzed by real-time quantitative RT-PCR using a Bio-Rad iCycler system (Bio-Rad, Hercules, CA). The specific primers for target genes in this study are listed in Table 1. The level of the housekeeping gene GAPDH was used as an internal control for the normalization of RNA quantity and quality differences among the samples.

### 2.9. Immunofluorescence

Immunofluorescent staining was performed as described [18]. Primary antibodies to CD68 and Ly6B (Abcam, Cambridge, MA) were used to detect the infiltration of macrophages and neutrophils, respectively, and primary antibodies to mouse anti-NOX2 (gp91; BD Company, Franklin Lakes, NJ) and rabbit anti-NeuN antibodies (Abcam) were used for the identification of NOX2 expression in neurons. Images were obtained by confocal laser-scanning microscopy using an LSM780 laser-scanning confocal microscope (Carl Zeiss, Germany) equipped with a Plan-Apochromat at 63x/1.4 objective. The total integrated density (IOD) for sections from different groups was analyzed by using the ImageJ analysis software (National Institutes of Health). The relative fluorescence intensity was used for statistical analysis.

### 2.10. Electron Spin Resonance (ESR) Detection of Superoxide (O_2_^•−^)

To detect O_2_^•−^ production, the electron spin resonance (ESR) assay was performed as described previously [19]. A spin trap, 1-hydroxy-3-methoxycarbonyl-2,2,5,5-tetramethylpyrrolidine (CMH; Enzo Life Sciences, USA), was prepared in the premixed Krebs Hepes Buffer (KHB) containing deferoxamine (100 *μ*mol/L; Sigma-Aldrich) and diethyldithiocarbamate (5 *μ*mol/L; Sigma-Aldrich) with a final concentration of 1 mmol/L. After being immediately homogenized, the mixtures containing brain tissue or cultured cells were loaded into glass capillaries and immediately analyzed for O_2_^•−^ formation kinetics in a Broker ESR A300 spectrometer (Bruker BioSpin Inc., Switzerland). The acquisition ESR parameters were as follows: biofield, 3488; sweep width, 500 G; microwave frequency, 9.78 GHz; microwave power, 20 mW; modulation amplitude, 3 G; modulation frequency, 100 kHz; sweep time, 83 s; and receiver gain, 2.0 × 10^3^. The ESR signal strength was recorded in arbitrary units, and the final results were expressed as the fold changes of the treated group compared to the sham-operated or control group. A synthetic cell-permeable mimetic of SOD, manganese (III) tetrakis (1-methyl-4-pyridyl) porphyrin pentachloride (MnTMPyP, 500 *μ*mol/L for cells and 5 mg/kg for animals; Sigma-Aldrich), was used as a positive control in this study.

### 2.11. Isolation of Mitochondria

Mitochondria and the cytoplasm were fractionated using a mitochondrial isolation kit (Pierce, Rockford, IL, USA) for brain homogenates or cultured cells by a density gradient centrifugation method according to the manufacturer's instructions as described [20]. All procedures were conducted at 4°C. In brief, the brain tissue or cultured cells were disrupted with a Dounce homogenizers in ice-cold mitochondrial isolation buffer manually by ten slow up-and-down strokes. Homogenates were centrifuged at 1000 ×g for 3 minutes at 4°C, and the postnuclear supernatant was centrifuged at 12,000 ×g for 15 minutes at 4°C. Mitochondrial pellet was resuspended with 50 mmol/L Tris-HCl (pH 7.4).

### 2.12. Western Blot Analysis

Brain homogenates, cultured cells, and isolated mitochondria and cytoplasm were examined by Western blot analysis [21]. A primary antibody to NOX2 was from BD Company (Franklin Lakes, NJ), and antibodies to AMPK, phosphor-AMPK (Thr172), and cytochrome C were from Cell Signaling Technology (USA). The membrane was reprobed with a primary antibody to GAPDH (Santa Cruz, USA) and COX IV (Cell Signaling Technology) to document the loading controls in whole cell homogenates and cytoplasm and mitochondria, respectively. MnTMPyP was used as a positive control for observing the expression level of NOX2.

### 2.13. ATP Content

The total ATP content in the brain tissue and cells was determined by using a luciferin-luciferase assay kit (Roche Diagnostics). In brief, homogenized brain tissues and cells were lysed in distilled water, and proteins were precipitated by addition of 5% HClO_4_. After centrifugation, an aliquot of the supernatant was adjusted to pH 7.7 by addition of saturated KHCO_3_ solution. After dilution, the ATP concentrations of the aliquots were determined by a full-wavelength Varioskan Flash according to the manufacturer's protocol [22].

### 2.14. Cell Culture and Treatments

The neuron-like rat pheochromocytoma cell line PC12 (Shanghai Cell Bank, Chinese Academy of Sciences) was cultured at 37°C in DMEM (Invitrogen, Gaithersburg, MD, USA) supplemented with 10% fetal bovine serum (Invitrogen), 100 U/mL penicillin, and 0.1 mg/mL streptomycin (Sigma-Aldrich). The model of oxygen-glucose deprivation (OGD) was induced by incubating cells in a hypoxic environment for 1 hour followed by 24 hours of reoxygenation as described previously [23]. The tested compounds or vehicle was added 2 hours prior to OGD treatment.

### 2.15. Measurement of Cell Viability and Death

Cell viability was determined by Cell Counting Kit-8 (CCK-8; Dojindo, Kumamoto, Japan) through the reagent WST-8 as described previously [24]. Treated cells were incubated in 10% CCK-8 that was diluted in normal culture medium at 37°C until the visual color conversion occurred. Optical density (OD) values were assessed at 450 nm using a full-wavelength Varioskan Flash. Cell death was determined by phycoerythrin (PE) annexin V in conjunction with 7-aminoactinomycin (7-AAD) staining by using the PE Annexin V Apoptosis Detection Kit (Becton-Dickinson, San Jose, CA) according to the manufacturer's protocol.

### 2.16. Intracellular Ca^2+^ Concentration Measurement

The levels of intracellular Ca^2+^ concentration ([Ca^2+^]_i_) were measured using fluorescent dye Fluo-3/AM (Invitrogen), which is a cell-permeable fluorescent indicator of intracellular Ca^2+^ and is nonfluorescent until it is hydrolyzed intracellularly and/or in the presence of Ca^2+^. Cells were incubated with 5 *μ*M Fluo-3/AM for 30 minutes at 37°C. The fluorescence intensity of Fluo-3/AM probes was analyzed with an excitation wavelength of 488 nm/an emission wavelength of 530 nm by flow cytometric analysis [25]. An L-type calcium channel inhibitor nimodipine (100 *μ*mol/L; Sigma-Alrich) was used as a control.

### 2.17. Measurement of the Mitochondrial Membrane Potential (MMP; Δ*Ψ*m)

The change in the MMP from isolated mitochondria was assessed using the fluorescent probe 5,5′,6,6′-tetrachloro-1,1′,3,3′-tetraethylimidacarbocyanine iodide (JC-1; Becton & Dickinson Company, USA). J-aggregates and JC-1 monomer were assessed by flow cytometry (Beckman, USA) with an excitation wavelength of 525 nm/490 nm and emission wavelength of 590 nm/530 nm, respectively. The mean fluorescence intensity ratio of JC-1 monomer (green fluorescence) to J-aggregates (red fluorescence) represents for the decrease in the mitochondrial membrane potential which is positively correlated with mitochondrial damage [26]. An inhibitor of mitochondrial permeability transition pore (mPTP), cyclosporine A (CsA, 50 *μ*mol/L; Sigma-Alrich), was used as a control.

### 2.18. Statistics

All data are expressed as means ± SD. The significance of the differences in mean values between and within multiple groups was examined by one-way analysis of variance (ANOVA) followed by Duncan's multiple range test (GraphPad Software Inc., USA). *P* < 0.05 was considered statistically significant.

## 3. Results

### 3.1. Primary Screening of Twin Compounds Containing HTMP and Carnitine Substructures in OGD-Induced Cell Death and in Experimental Ischemic Stroke

**Figure 2:**
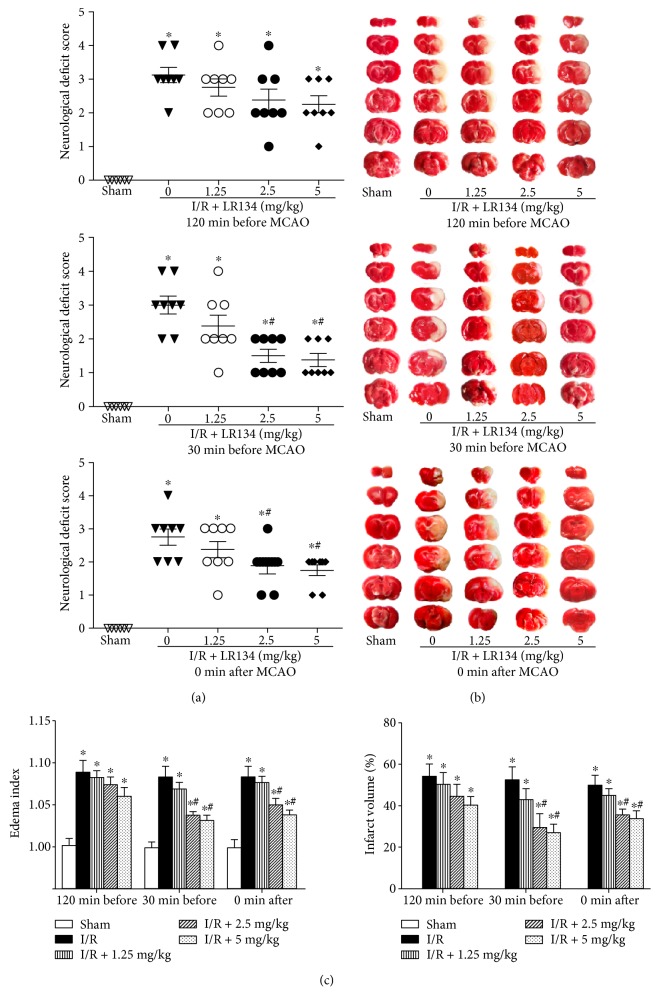
Primary time and dose screening of the compound LR134 in experimental ischemic stroke. (a) Neurological deficit scores showing neurobehavioral manifestations in LR134-treated cerebral ischemic rats at different administration times. (b) Representative photographs of 2,3,5-triphenyltetrazolium chloride (TTC) staining in LR134-treated cerebral ischemic rats at different administration times. (c) The cerebral edema index and calculated infarct volume in different groups of rats. ^∗^*P* < 0.05 versus sham-operated (sham) group, ^#^*P* < 0.05 versus ischemia reperfusion- (I/R-) vehicle group (*n* = 8).

We first evaluated the protective effects of HTMP-carnitine ester twin compounds on PC12 cells under OGD condition using the CCK-8 assay, and the concentration for 50% of the maximal effect (EC50) was calculated by plotting the dependence between drug concentration and percentage of protection on OGD-induced cell death [27]. As shown in Table 2, among the ten candidate compounds, the EC50 of four candidates (LR134, 137, 140, and 143) was less than 100 nmol/L and the compound LR134 (with the minimum EC50) was selected for further *in vivo* dose and time screening study. We applied the compounds 120 minutes and 30 minutes before MCAO and immediately (0 minute) after MCAO. Compared with the vehicle-treated I/R group, the neurobehavioral manifestations (Figure 2(a)), infarct volume), and edema index (Figure 2(b) and 2(c)) were significantly reduced in the LR134-administrated group at 30 minutes before MCAO and 0 minute after MCAO, which is consistent with the pharmacokinetics results showing that after a single intravenous administration, the peak time of the compound LR134 in the rats' brain is about 30 minutes. As for dose screening, 2.5 and 5 mg/kg LR134-treated groups showed significant neuroprotection as compared with the vehicle-treated group. Therefore, we chose the minimal tested protective molar doses (2.5 mg/kg of LR134, 6.82 *μ*mol/kg) and 30 minutes before MCAO showing more obvious protective effects for further studies. Meanwhile, administration of LR134 had no effects on cerebral function under sham-operated states (Figure S2 in Supplementary Materials).

### 3.2. Both LR134 and LR143 Protected against Cerebral Ischemia Reperfusion (I/R) Injury in Rats

**Figure 3:**
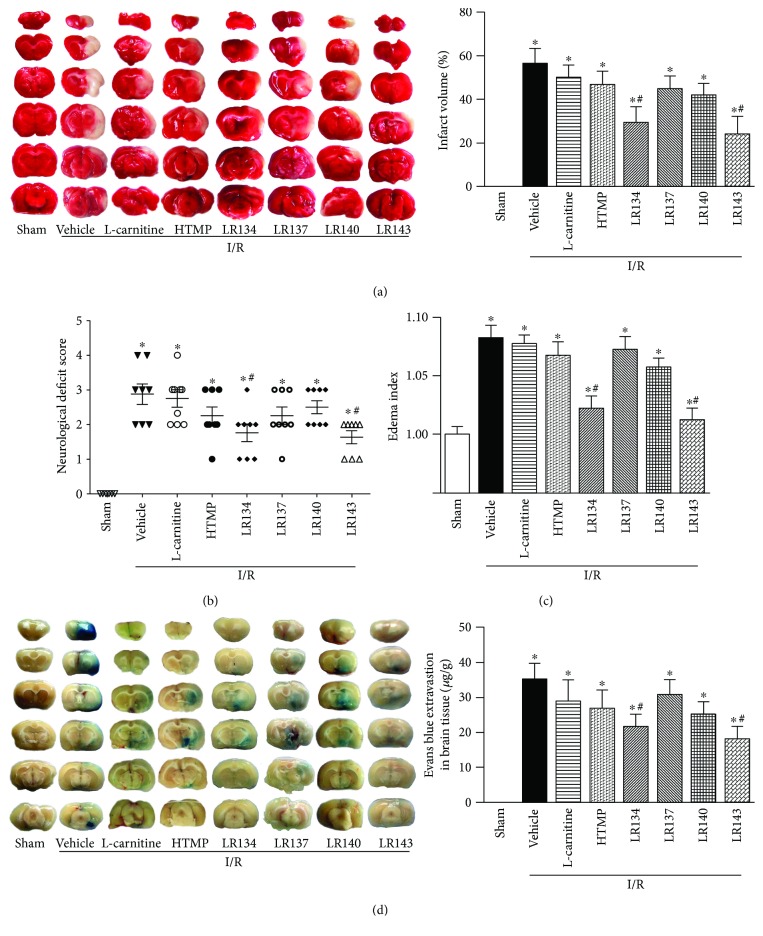
Both LR134 and LR143 protected against cerebral ischemia reperfusion (I/R) injury in rats. (a) Representative photographs of TTC staining and calculated infarct volume showing the effects of candidate 2-hydroxymethyl-3,5,6-trimethylpyrazine-carnitine ester twin compounds in rats with cerebral I/R injury. (b) Neurological deficit scores in ischemic rats with different treatments. (c) The cerebral edema index in ischemic rats with different treatments. (d) Representative images of brain slices after Evans blue injection and calculated Evans blue intensity in ischemic rats with different treatments. ^∗^*P* < 0.05 versus sham group, ^#^*P* < 0.05 versus I/R-vehicle group (*n* = 8).

At the equal molar dose (6.82 *μ*mol/kg) and 30 minutes before MCAO, we found that besides the compound LR134, administration of the compound LR143 also decreased infarct size (Figure 3(a)), neurological deficit (Figure 3(b)), edema index (Figure 3(c)), and BBB permeability (Figure 3(d)), while the compounds LR137 and LR140 had no significant protective effects on experimental ischemic stroke at the same dose. However, the precursors of the compounds, L-carnitine and HTMP, had no significant protective effects on cerebral I/R injury at the same dose.

### 3.3. LR134 and LR143 Ameliorated Neuronal Injury in Rats with Ischemic Stroke

**Figure 4:**
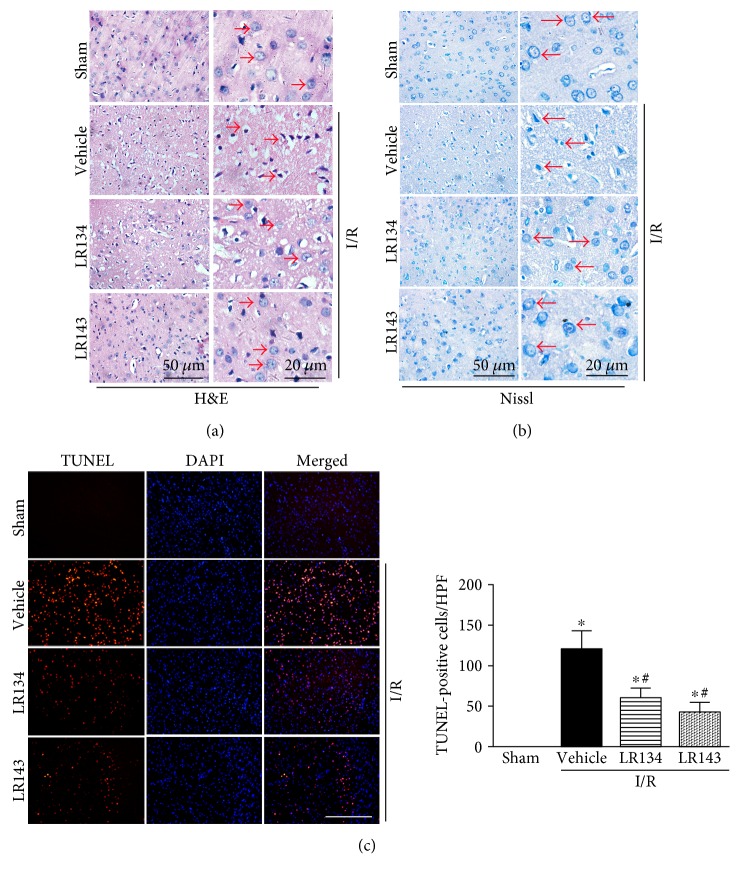
LR134 and LR143 ameliorated neuronal injury in rats after cerebral ischemia reperfusion (I/R) injury. (a) Representative photomicrographs of hematoxylin and eosin (H&E) in the cerebral cortex of rats after I/R injury. (b) Cresyl violet (Nissl) staining in the cerebral cortex of rats after cerebral I/R injury. (c) In situ terminal deoxynucleotidyl transferase-mediated uridine triphosphate nick end labeling (TUNEL, red) assays were performed to assess cell death. Nuclei were stained with 4′,6-diamidino-2-phenylindole (DAPI, blue); the TUNEL-positive cells indicated the quantitative assessment of cell death (numbers per high power field). Red arrowheads indicated neuronal cells. ^∗^*P* < 0.05 versus sham group, ^#^*P* < 0.05 versus I/R-vehicle group (*n* = 8).

To further assess the neuroprotective effects of these two candidate compounds in ischemia-evoked neuronal injury, H&E staining was used to examine morphological features of injured neurons in the cerebral cortex, cresyl violet (Nissl staining) was used to examine the surviving cells of neurons, and TUNEL staining was used to assess the neuronal cell death. We found that I/R induced severe neuronal necrosis (Figure 4(a)), a significant reduction in Nissl substance (Figure 4(b)) indicating the loss of surviving neurons, and an increased number of TUNEL-positive cells (Figure 4(c)). However, administration of LR134 and LR143 significantly ameliorated neuronal injury.

### 3.4. LR134 and LR143 Reduced Inflammatory Responses and NOX2-Mediated Oxidative Stress in Rats after Cerebral Ischemia Reperfusion (I/R) Injury

**Figure 5:**
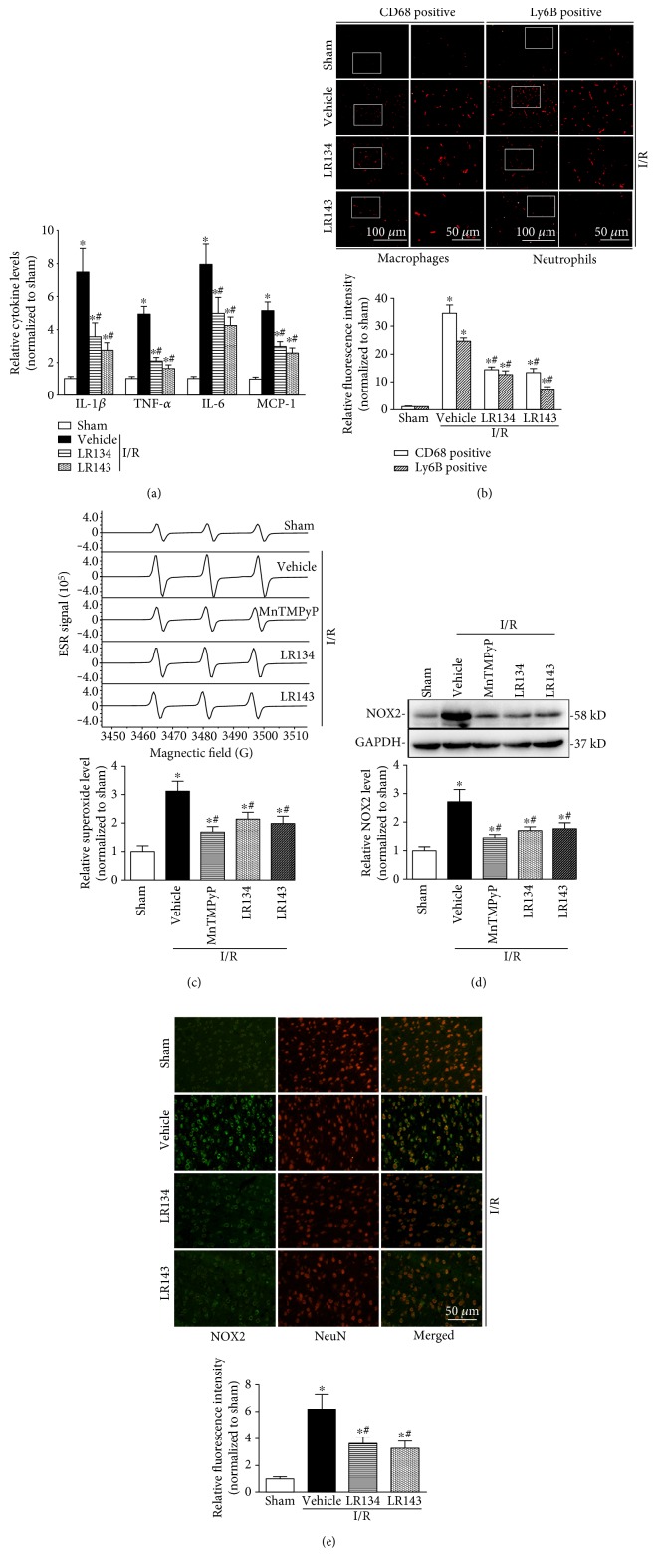
LR134 and LR143 reduced inflammatory responses and NOX2-mediated oxidative stress in rats after cerebral ischemia reperfusion (I/R) injury. (a) The mRNA levels of proinflammatory mediators including IL-1*β* (interleukin-1*β*), tumor necrosis factor-*α* (TNF-*α*), IL-6, and MCP-1 (monocyte chemoattractant protein-1) were measured by real-time RT-PCR analysis. (b) Representative sections and quantitative analysis of brain stained for macrophage (CD68 positive) and neutrophil (Ly6B positive) infiltration in the brain from different groups of rats. (c) Representative electron spin resonance (ESR) spectrographs and summarized data showing the relative levels of superoxide (O_2_^•−^) production in the brain from different groups of rats. (d) Representative Western blot gel documents and summarized data showing NOX2 levels in different groups of rats. (e) Representative brain sections and quantitative analysis showing the upregulation of NOX2 in neurons after cerebral ischemia/reperfusion by immunofluorescence analysis. ^∗^*P* < 0.05 versus sham-operated (sham) group, ^#^*P* < 0.05 versus I/R-vehicle group (*n* = 8).

As shown in Figures 5(a) and 5(b), cerebral I/R markedly enhanced the mRNA levels of proinflammatory mediators consistent with the increased Ly6B-positive neutrophils and CD68-positive macrophage infiltration, which was significantly attenuated by LR134 and LR143. Considering that NADPH oxidase-mediated oxidative stress significantly contributes to cerebral I/R injury, we further measured O_2_^•−^ production and the expression of NOX2, a major functional subunit of NADPH oxidase during ischemic stroke. The result showed that the O_2_^•−^ level was markedly increased in the brain after MCAO (Figure 5(c)), which was consistent with the increased expression of NOX2 (gp91*^phox^*) (Figure 5(d)). The results from coimmunofluorescence staining for NOX2 and a neuronal marker NeuN indicated the upregulation of NOX2 in neurons (Figure 5(e)). Pretreatment of LR134 and LR143 markedly decreased O_2_^•−^ production and NOX2 expression induced by I/R injury.

### 3.5. LR134 and LR143 Improved Energy Metabolism and Mitochondrial Dysfunction

**Figure 6:**
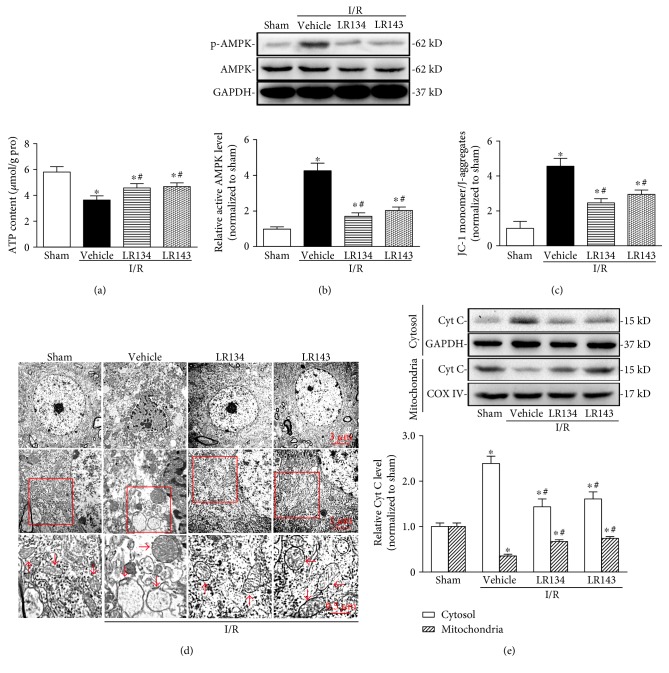
LR134 and LR143 improved energy metabolism and mitochondrial dysfunction. (a) ATP contents in brain tissues from different groups of rats at 24 hours of reperfusion after MCAO. (b) Representative Western blot gel documents and summarized data showing the levels of activated AMPK in the brain from different groups of rats. (c) Mitochondrial membrane potential was detected with the fluorescent probe JC-1 staining and presented by the ratio of JC-1 monomer to J-aggregates followed by flow cytometric analysis. (d) Transmission electron microscope analysis showing the images of neurons and mitochondria from different groups of rats. (e) Representative Western blot gel documents and summarized data showing the cytochrome C release from mitochondria to the cytosol. ^∗^*P* < 0.05 versus sham-operated group, ^#^*P* < 0.05 versus I/R-vehicle group (*n* = 8).

By the measurement of ATP content in dissociated brain tissues, we found that pretreatment of the compounds LR134 and LR143 significantly improved energy metabolism in experimental ischemic stroke (Figure 6(a)). Since the AMP-activated protein kinase (AMPK) is a critical energy sensor in all types of cells, we then measured the activation of AMPK presented by the ratio of the phosphorylation form of AMPK on Thy 172 to total AMPK. It was found that LR134 and LR143 remarkably reduced AMPK activity (Figure 6(b)). Considering the importance of mitochondrial dysfunction in the ischemic stroke, we then evaluated the effects of LR134 and LR143 on both structure and function of brain mitochondria. The significant decrease in the mitochondrial membrane potential (MMP) indicated mitochondrial damages in I/R groups, while pretreatment of the compounds LR134 and 143 ameliorated mitochondrial damages (Figure 6(c)). The ultrastructural analysis revealed the presence of abnormally and irregularly swelling mitochondria with shortening and disintegrating cristae, as well as the vacuole in ischemic brains. Pretreatment with LR134 and LR143 improved mitochondrial structure as evidenced by the presence of regularly rounded appearance with increased number of elongated cristae (Figure 6(d)). Subsequently, the release of cytochrome C from mitochondria into the cytoplasm was also observed to detect the mitochondrial function. As shown in Figure 6(e), Western blot showed elevated levels of cytochrome C in the cytosol and concomitantly lower levels in mitochondria of ischemic brain tissues, indicating that this apoptogenic factor was released from mitochondria to the cytoplasm. Moreover, LR134 and LR143 inhibited the release of cytochrome C into the cytoplasm.

### 3.6. Compounds LR134 and LR143 Improved Cellular Viability via Mitochondrial Protection in PC12 Cells under OGD Condition

**Figure 7:**
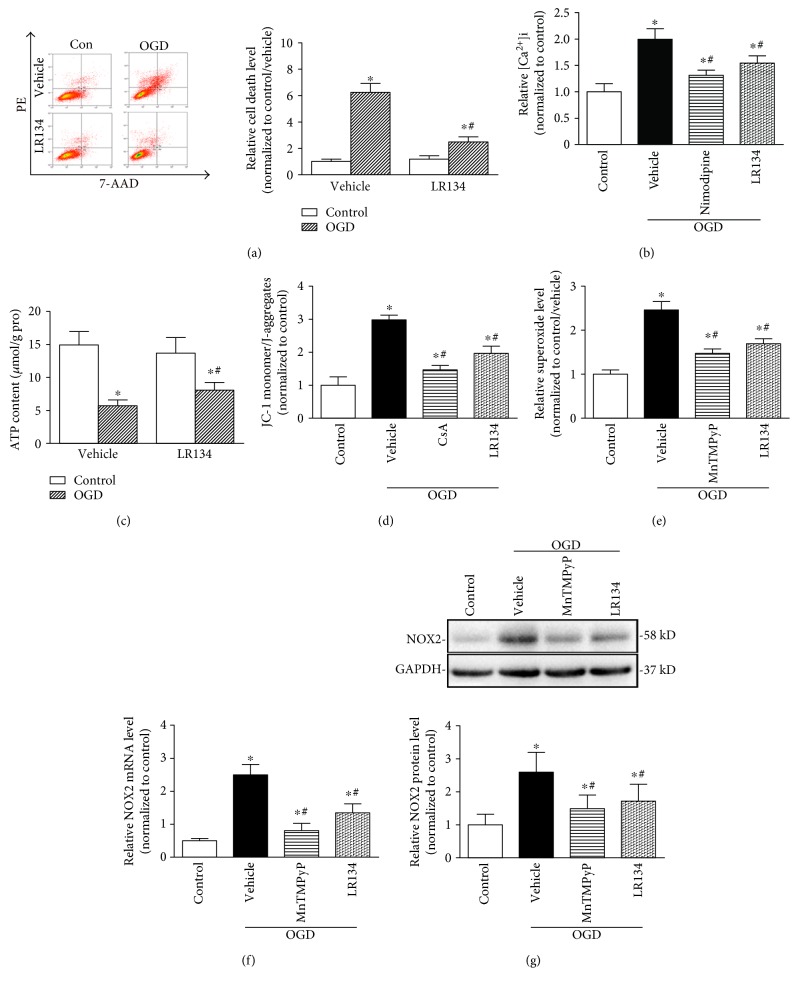
LR134 protected against cell injury in PC12 cells under OGD condition. (a) The quantified data showing cell death determined by flow cytometric analysis. (b) The intracellular Ca^2+^ concentration ([Ca^2+^]i) was measured with a calcium indicator dye, Fluo-3/AM. (c) ATP contents in PC12 cells with different treatments. (d) Mitochondrial membrane potential was detected with the fluorescent probe JC-1 staining and presented by the ratio of JC-1 monomer to J-aggregates followed by flow cytometric analysis. (e) Summarized data showing the relative levels of superoxide (O_2_^•−^) production in PC12 cells with different treatments. (f) The relative NOX2 mRNA levels in PC12 cells under OGD condition. (g) Representative Western blot gel documents and summarized data showing the relative NOX2 protein levels in PC12 cells with different treatments. ^∗^*P* < 0.05 versus control-vehicle group, ^#^*P* < 0.05 versus OGD-vehicle group (*n* = 6).

We then performed *in vitro* studies to confirm the protective effects of the candidate twin compounds. We provided experiments for the screening of the optical concentration and reoxygenation time for further study. The results showed that incubated cells with a series of concentrations of LR134 (10^−9^ to 10^−6^ mol/L) resulted in significant inhibition of OGD-induced cell death for 3 to 24 hours after reoxygenation, with no significant adverse effects on normal cell growth (Figure S3 in Supplementary Materials). We then chose the concentration of 10^−7^ mol/L and reoxygenation time of 24 hours for further studies. Furthermore, OGD significantly induced cell death measured by flow cytometric analysis, which can be attenuated by LR134 (Figure 7(a)). Mechanically, LR134 inhibited the intracellular calcium release (Figure 7(b)), ATP content (Figure 7(c)), and mitochondrial membrane potential loss (Figure 7(d)). We further found that LR134 inhibited OGD-induced O_2_^•−^ production (Figure 7(e)), as well as NOX2 mRNA (Figure 7(f)) and protein levels (Figure 7(g)) in PC12 cells under OGD condition. Similar results were also found in LR143-treated cells (data not shown).

## 4. Discussion

**Figure 8:**
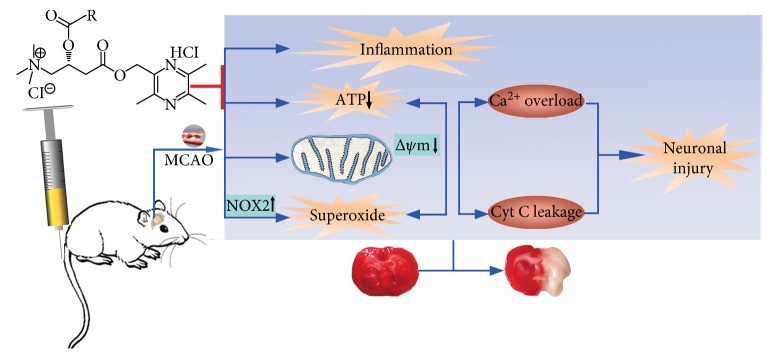
Schematic depicting therapeutic potential and related mechanisms of novel twin compounds containing tetramethylpyrazine and carnitine substructures in experimental ischemic stroke.

Although studies have indicated that both the precursor compounds L-carnitine and TMP derivatives exhibited neuroprotective effects [5, 28], the individual disadvantages limit their therapeutic application for the treatment of ischemic stroke. In this study, we identified for the first time that two novel twin compounds containing HTMP and carnitine substructures protected against cerebral I/R injury, which is associated with pleiotropic potency including anti-inflammation and antioxidant and maintenance of mitochondrial function (Figure 8).

By a combination of the neuroprotective effects of TMP derivatives and the drug delivery property of carnitine esters, we designed and synthesized a series of novel twin compounds containing HTMP and carnitine substructures. First, we screened the protective effects of ten synthesized target compounds on the viability of PC12 cells under OGD condition *in vitro* and in an experimental animal ischemic stroke model; we found that both compounds LR134 and LR143 with low EC50 exhibited significant neuroprotection against cerebral I/R injury in rats by reducing cerebral infarct volume and edema, improving neurological function as well as blood-brain barrier integrity. The neuroprotective effects were further confirmed by significant improvement of morphological features and the viability of impaired neurons by TUNEL and flow cytometric analyses.

The inflammatory responses accompanying stroke are recognized to contribute to secondary ischemic injury including induced microglial and astrocyte activity and increased the production of proinflammatory mediators; therefore, strategies targeting inflammation represent a promising therapeutic option [29]. Our results further support the neuroprotective effects of two candidate compounds via effectively suppressing ischemia-associated inflammation as evidenced by decreased mRNA levels of proinflammatory mediators and infiltration of inflammatory cells.

Apart from inflammatory responses, reperfusion after stroke attack accounts for a burst of reactive oxygen species (ROS) generation and results in oxidative stress, which plays a pivotal role in the pathogenesis of stroke [30]. NADPH oxidase is a major superoxide-producing enzyme in the brain cells. Among the major functional subunits of NADPH oxidase involved in the pathogenesis of stroke, NOX2 is one of the most important members mediating cerebral ischemic injury [31]. Consistent with the antioxidative roles of L-carnitine [4] and TMP [32], we found that LR134 and LR143 reduced NOX2 expression together with superoxide production. An increasing number of evidence have indicated that activation of the immune system as a result of disturbances in the redox state of cells seems to contribute to brain damage. Although we did not attempt to investigate why LR134- and LR143-treated animals potentially recruit less inflammatory cells, a recent study has found that endothelial cell NOX2-derived ROS production can induce aortic dissection by increased endothelial cell activation, leading to inflammatory cell recruitment throughout the aortic wall [33]. Other studies have also shown that inhibition of NADPH oxidase expression and activity prevented homocysteine-induced infiltration of macrophages and T-cells into the glomeruli, probably by mediating NLRP3 inflammasome activation [34]. The exact mechanisms in our study need to be further investigated.

In addition, the disruption of blood flow to brain tissue during stroke attack causes a metabolic crisis, which directly results in acute reduced energy supply. Meanwhile, neurons are particularly vulnerable to ischemic injury because they require a large amount of energy for maintaining normal neurotransmission; any impairment of blood flow leads to a rapid depletion of the energy in affected areas of the brain with ensuing neuronal damage [35]. To test the potential role of candidate compounds on energy failure after ischemia, we directly measured ATP content in dissociated brain cells and found that administration of LR134 and LR143 increased the ATP levels which were reduced in the ischemic hemisphere by MCAO, indicating that the recovery of cellular energy failure after ischemic insult by LR134 and LR143 helps rescue neurons to avoid irreversible injury. At molecular levels, we further detected the activation of 5′-adenosine monophosphate kinase (AMPK), a key sensor of cellular energy status that plays a vital role in the regulation of neuronal survival during ischemic stroke. Despite some reports suggesting the protective role of AMPK during early and transient energy depletion [36], an increasing number of studies recognized the detrimental role of prolonged AMPK activation which results in neuronal apoptosis via the activation of cell death machinery; therefore, pharmacological inhibition or gene deletion of AMPK is neuroprotective in stroke [37]. Our results showed a significant increase in the phosphorylation state of AMPK after cerebral I/R, which was blocked by pretreatment of the compounds LR134 and LR143, indicating that the protective role of LR134 and LR143 in stroke is associated with the inhibition of AMPK activation.

It should be noted that during stroke, the diminished brain blood supply may consequently result in subcellular damage other than cellular damage. Mitochondria not only functioned as “energy houses” but also serve as the main source of enhanced ROS during the course of ischemic brain injury. Studies have demonstrated that cerebral I/R injury is at least in part initiated by the mitochondrial disorder [38]. In this study, we further tested whether the candidate twin compounds directly act on mitochondria. In view of the importance of abnormal mitochondrial membrane potential, with the energy impairments and intracellular calcium overload in the progression of mitochondrial dysfunction being major properties [39], we measured the depolarization of Δ*Ψ*m and intracellular calcium content in PC12 cells injured by OGD condition and found that both twin compounds are associated with the mitochondrial improvement pathway by modulating calcium and mitochondrial membrane potential. The results of *in vivo* studies are in agreement with the *in vitro* mitoprotective effects of twin compounds LR134 and LR143 in MCAO-injured rat brain mitochondria, as evidenced by inhibiting the loss of mitochondrial membrane potential, improving mitochondrial ultrastructure and decreasing the release of cytochrome C from mitochondria to the cytoplasm, a consequence of the mitochondrial membrane permeability transition [40].

In conclusion, this study provides direct evidence showing that the novel twin compounds containing tetramethylpyrazine and carnitine substructures protect against cerebral injury in experimental stroke, which is associated with reduced inflammatory responses and oxidative stress, as well as with the protection of mitochondrial structure and function, suggesting that modulation of these chemical structures may be an innovative therapeutic strategy for treating patients with stroke.

## Figures and Tables

**Table 1:** Primer pairs of target genes used for real-time RT-PCR in this study.

Genes	Accession number	Forward	Reverse
IL-1*β*	NM_031512.2	CCAAGCCCTTGACTTGGGCTGTC	TGGGTCCTCATCCTGGAAGCTCC
TNF-*α*	XM_008772775.1	CCACCACGCTCTTCTGTCTA	TTTGCTACGACGTGGGCTAC
IL-6	NM_012589.2	CCGGAGAGGAGACTTCACAGAG	CAGTGCATCATCGCTGTTCATAC
MCP-1	NM_031530.1	CTGGGCCTGTTGTTCACAGTTGC	CTTTGGGACACCTGCTGCTGGTG
NOX2	NM_023965.1	TGTGGCTGTGATAAGCAGGA	TCCCACTAACATCACCACCT
GAPDH	NM_017008.4	TGCATCCTGCACCACCAACTGC	ACAGCCTTGGCAGCACCAGTGG

**Table 2:** Primary screening of neuroprotection and calculated median effective concentration (EC50) of 2-hydroxymethyl-3,5,6-trimethylpyrazine-carnitine ester twin compounds on oxygen-glucose deprivation- (OGD-) injured PC12 cells.

Compound	R	EC50 (nmol/L)
LR102	CH_2_CH_3_	>100
LR109	CH=CH-Ph	>100
LR120	CH_3_	>100
LR134	CH_2_CH_2_CH_3_	43.92 ± 10.93
LR137	CH(CH_3_)_2_	75.23 ± 13.36
LR140	(CH_2_)_3_CH_3_	50.88 ± 6.30
LR143	CH_2_CH(CH_3_)_2_	58.03 ± 11.36
LR146	(CH_2_)_4_CH_3_	>100
LR149	(CH_2_)_7_CH_3_	>100
LR152	(CH_2_)_7_CH=CH(CH_2_)_7_CH_3_	>100

## Supplementary Materials

Supplementary materials Figure S1: The regional cerebral blood flow (rCBF) in the middle cerebral artery region was monitored by laser doppler flowmetry. Figure S2: The effects of Compound LR134 on stroke outcomes under sham-operated and cerebral ischemia reperfusion (I/R) states. Figure S3: Cell viability was evaluated by CCK-8 (WST-8) assay in PC12 cells under OGD condition with different concentrations (A) or time treatments (B) of compound LR134.
