# Supplementary material for: Therapeutic Potential of Novel Twin Compounds Containing Tetramethylpyrazine and Carnitine Substructures in Experimental Ischemic Stroke
Source: Oxid Med Cell Longev. 2017 Dec 13;2017:7191856. doi: 10.1155/2017/7191856 (PMC5745738; doi:10.1155/2017/7191856)
Supplement: Supplementary materials — Figure S1: The regional cerebral blood flow (rCBF) in the middle cerebral artery region was monitored by laser doppler flowmetry. Figure S2: The effects of Compound LR134 on stroke outcomes under sham-operated and cerebral ischemia reperfusion (I/R) states. Figure S3: Cell viability was evaluated by CCK-8 (WST-8) assay in PC12 cells under OGD condition with different concentrations (A) or time treatments (B) of compound LR134. [file 7191856.f1.zip › 7191856.f2.pdf]

SUPPLEMENTARY MATERIALS

Therapeutic potential of novel twin compounds containing tetramethylpyrazine and carnitine substructures in experimental ischemic stroke

Ziying Wang[1,2], Zhuanli Zhou[1], Xinbing Wei[1], Mingwei Wang[1], Bi-Ou Wang[1], Yanan Zhang[1], Xiaoting He[1], Yu Sun[1], Xiaojie Wang[1], Mingcheng Sun[1],Yan Zhang[1], Xiaowei Gong[2], and Fan Yi[1]*

[1]Department of Pharmacology, School of Basic Medical Science, Shandong University, Jinan, 250012, China
[2]Key Laboratory of Neuroprotective Drug Discovery of Shandong Province, 250012, China.

Running title:
HTMP-carnitine ester twin compounds protect against stroke.

*Send Correspondence and Reprint Requests to:

Fan Yi, Ph.D
Professor
Department of Pharmacology
School of Basic Medical Science
Shandong University
44#, Wenhua Xi Road,
Jinan, Shandong, 250012, China
Phone: 86-0531-88382616
Fax: 86-0531-88382616
E-mail: fanyi@sdu.edu.cn

Materials and Methods

Chemical characterization of tested compounds: The chemical structures of candidate 2-hydroxymethyl-3, 5, 6-trimethylpyrazine (HTMP)-carnitine ester twin-compounds were confirmed by ^{1}H nuclear magnetic resonance, infra-red spectrum and high resolution mass spectra detections as described previously [1]. Infrared (IR) spectra were recorded in the range of 4000~600 cm^{-1} using a Nicolet Nexus 470FT-Spectrometer with KBr disks. ^{1}H NMR spectra was determined in D_2O on a Bruker 600 MHz spectrometer with TMS as an internal standard. Electrospray ionization mass spectrometry (ESI-MS) was performed on an API 4000 spectrometer. Elemental analyses were carried out on an Elementar Elvario III C, H, and N elemental analyzer.

Regional cerebral blood flow by laser doppler flowmetry: The anesthetized animal was placed in a stereotaxic apparatus. After the midline scalp was shaved, disinfected and sliced, the galea and periosteum overlying the parietal bone were swept. The regions of the cerebral cortex supplied by middle cerebral artery (at a dimension 2mm posterior and 6mm lateral from the bregma) were thinned by a high speed dental drill until the inner cortical layer of bone was encountered. A flexible 0.5 mm fiber-optic extension to needle probe was fixed on the ipsilateral side of the superior portion over the ischemic cortex. The cerebral blood flow (CBF) signals were recorded by laser doppler flowmetry (Perimed, Jarfalla, Sweden) and analyzed using AcqKnowledge 4.0 software. The laser doppler signals is expressed as microvacular blood perfusion units (PU). In this study, CBF was measured at 3 time points: 1. before MCAO, 2. after MCAO and 3. after reperfusion. During the CBF measurement, animals were kept anesthesia and the body temperature was maintained at 37◦C [2].

References

[1] S. Sultan, M. Z. Noor, H. Anouar el et al., "Structure and absolute configuration of 20beta-Hydroxyprednisolone, a biotransformed product of predinisolone by the marine endophytic fungus Penicilium lapidosum," Molecules, vol. 19, no. 5, pp. 13775-13787, 2014.

[2] T. Liu, B. Xiang, D. Guo et al., "Morroniside promotes angiogenesis and further improves microvascular circulation after focal cerebral ischemia/reperfusion," Brain Research Bulletin, vol. 127, pp. 111-118, 2016.

Results

Chemical structure of compound LR134:

(R)-4-((3,5,6-trimethylpyrazin)-2-methoxy)-2-(butyryloxy)-N,N,N-trimethyl-4-oxobutan-1-aminium chloride.

^{1}H NMR (300 Hz, D_2O): 0.75 (3H, t, CH_3CH_2-, J=15); 1.39-1.55 (2H, m, CH_3CH_2-); 2.22-2.30 (2H, m, -CH_2CH_2COO-); 2.54 (3H, s, $CH_3C(N)$-); 2.57 (3H, s, $CH_3C(N)$-); 2.60 (3H, s, $CH_3C(N)$-); 2.87-2.90 (2H, m, -CH_2COO-); 3.11 (9H, s, $(CH_3)3N$-); 3.58 (2H, d, d, -NCH_2-, J=6, J=14.1); 5.33 (2H, s, -$COOCH_2$-); 5.57 (1H, m, -NCH_2CH-)

IR (KBr plate): 2966 (γCH), 1742 (γC=O), 1627 (γC=C), 1513, 1413 (δCH2, CH3), 1175 (νC-N), 681, 695, 715, 972 (aromatic ring).

ESI-MS (m/z): 366.5 $[M^+ H]^+$.

Chemical structure of compound LR137:

(R)-4-((3,5,6-trimethylpyrazin)-2-methoxy)-2-(isobutyryloxy)-N,N,N-trimethyl-4-oxobutan-1-aminium chloride.

^{1}H NMR(300 Hz, D_2O): 2.46-2.49 (1H, m, $(CH_3)_2CH$-); 2.52 (3H, s, $CH_3C(N)$-); 2.54 (3H, s, $CH_3C(N)$-); 2.55 (3H, s, $CH_3C(N)$-); 2.84-2.88 (2H, m, -CH_2COO-); 3.09 (9H, s, $(CH_3)3N$-); 3.57 (2H, d, d, -NCH_2-, J=4.5, J=13.2); 5.32 (2H, s, -$COOCH_2$-); 5.58 (1H, m, -NCH_2CH-)

IR (KBr plate): 2975 (γ CH), 1739 (γC=O), 1628 (γ C=C), 1514 (γ Ar-C), 1472 (δ CH2, CH3), 1178 (νC-N), 698, 7168, 939 (aromatic ring).

ESI-MS (m/z): 366.2 $[M^+H]^+$.

Chemical structure of compound LR140:

(R)-4-((3,5,6-trimethylpyrazin)-2-methoxy)-2-(pentanoyloxy)-N,N,N-trimethyl-4-oxobutan-1-aminium chloride.

^{1}H NMR (300 Hz, D_2O): 0.76-0.81 (3H, m, CH_3CH_2-); 1.14-1.24 (2H, m, -CH_3CH_2-); 1.44-1.52 (2H, m, -$CH_2CH_2CH_2$-); 2.26-2.30 (2H, m, -CH_2CH_2COO-); 2.54 (3H, s, $CH_3C(N)$-); 2.58 (3H, s, $CH_3C(N)$-); 2.60 (3H, s, $CH_3C(N)$-); 2.86-2.89 (2H, m, -$CHCH_2COO$-); 3.11 (9H, S, (CH_3) 3N-); 3.59 (2H, d, d, -NCH_2-, J=4.8, J=14.4); 5.33 (2H, s, -$COOCH_2$-); 5.57 (1H, m, -NCH_2CH-)

IR (KBr plate): 2961 (γ CH), 1728 (γC=O), 1629 (γC=C), 1514 (γAr-C), 1480, 1391 (δ CH2, CH3), 1182 (vC-N), 698, 715, 871, 935 (aromatic ring).

ESI-MS (m/z): 380.5 $[M^{+}H]^{+}$.

Chemical structure of compound LR143: (R)-4-((3,5,6-trimethylpyrazin)-2-methoxy)-2-(3-methylbutanoyloxy) -N,N,N- trimethyl-4- oxobutan-1-aminium chloride.

^{1}H NMR (300 Hz, D_2O): 0.74-0.87 (6H, m, $(CH_3)_2CH$-); 1.78-1.86 (1H, m, $(CH_3)_2$ CH-); 2.15 (2H, d, -CH_2COOCH-); 2.57 (3H, s, $CH_3C(N)$-); 2.65 (3H, s, CH_3 C(N)-); 2.70 (3H, s, $CH_3C(N)$-); 2.86-2.90 (2H, t, -CH_2COOCH_2-); 3.11 (9H, S, $(CH_3)_3N$-); 3.61 (2H, d, d, -NCH_2-, J=7.2, J=14.7); 5.33 (2H, s,-$COOCH_2$-); 5.56-5.62 (1H, m,-NCH_2CH-)

IR (KBr plate): 2961 (γ CH), 1742 (γC=O), 1627 (γC=C), 1514 (γAr-C), 1467, 1413 (δ CH2, CH3), 1180 (vC-N), 608, 715, 938 (aromatic ring).

ESI-MS (m/z): 380.6 $[M^{+}H]^{+}$.

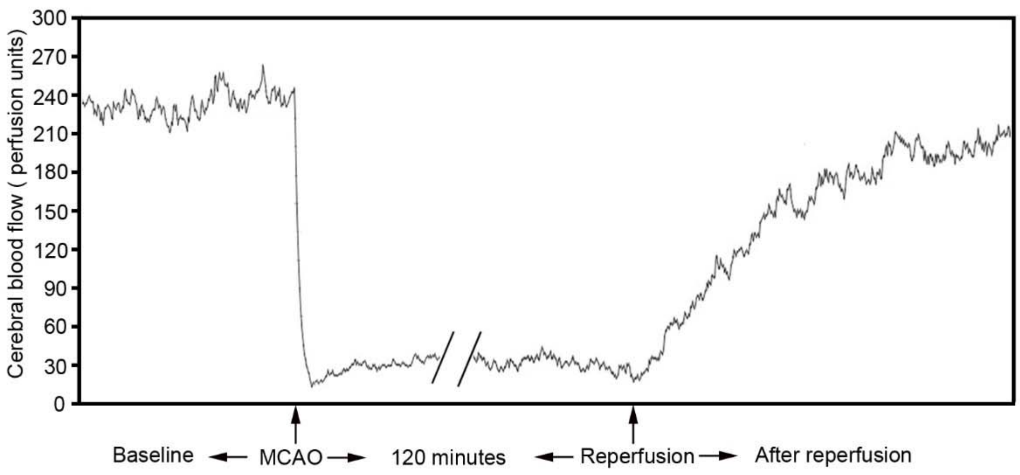

Figure S1 The regional cerebral blood flow (rCBF) in the middle cerebral artery region was monitored by laser doppler flowmetry.

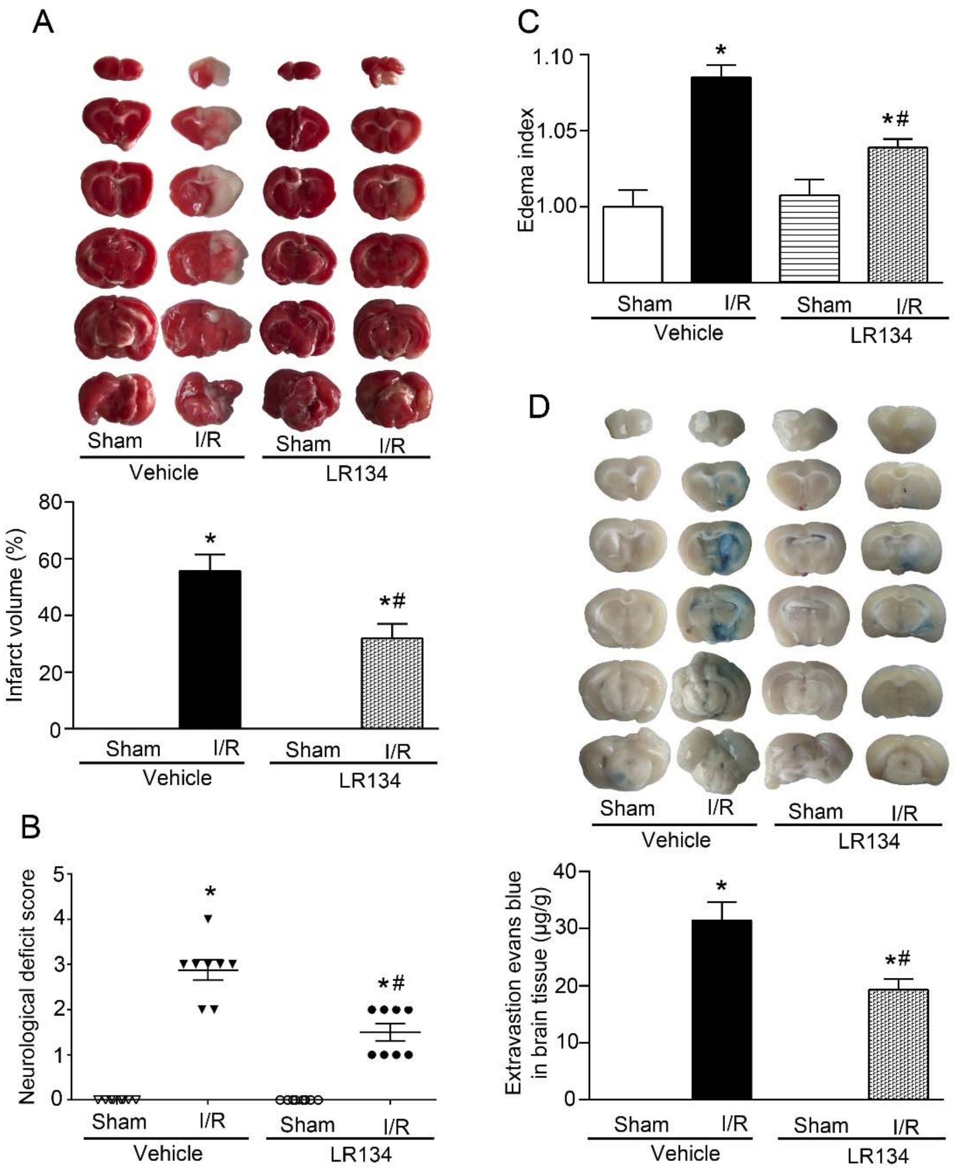

Figure S2. The effects of Compound LR134 on stroke outcomes under sham-operated and cerebral ischemia reperfusion (I/R) states. (A) Representative photographs of TTC staining and calculated infarct volume in LR134 treated rats under different conditions. (B) Neurological deficit scores in LR134 treated rats under different conditions. (C) Cerebral edema index in in LR134 treated rats under different conditions. (D) Representative images of brain slices after Evans blue injection and calculated Evans blue intensity in LR134 treated rats under different conditions. *P < 0.05 versus sham-vehicle group, $^{\#}P$<0.05 versus I/R-vehicle group (n=8).

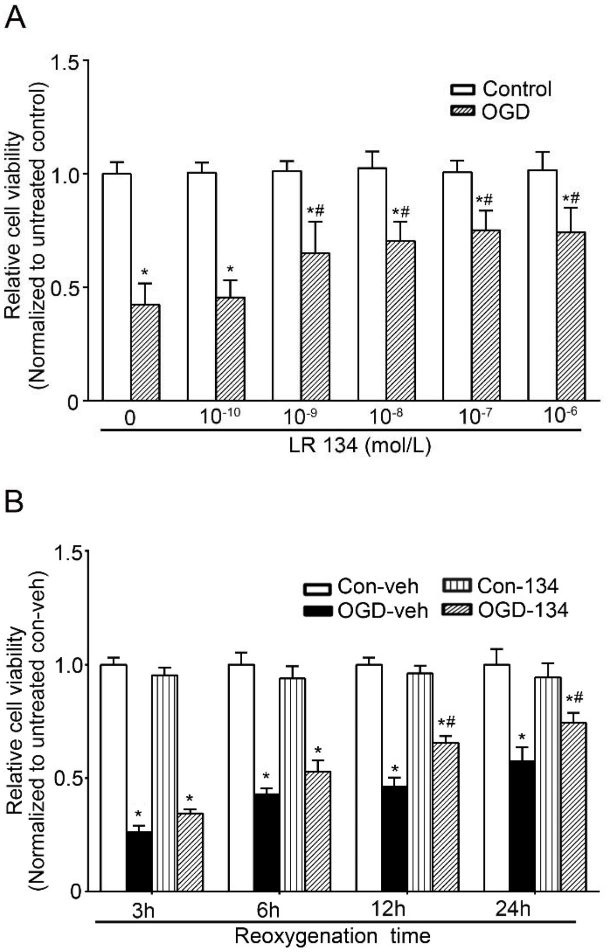

Figure S3 Cell viability was evaluated by CCK-8 (WST-8) assay in PC12 cells under OGD condition with different concentrations (A) or time treatments (B) of compound LR134. *P<0.05 vs. untreated control (vehicle) group, #P<0.05 vs. untreated OGD (vehicle) group (n=6).
